# Cellular Mechanisms Underlying the Laxative Effect of Flavonol Naringenin on Rat Constipation Model

**DOI:** 10.1371/journal.pone.0003348

**Published:** 2008-10-03

**Authors:** Zi-Huan Yang, Hai-Jie Yu, Ao Pan, Jian-Yang Du, Ye-Chun Ruan, Wing-Hung Ko, Hsiao-Chang Chan, Wen-Liang Zhou

**Affiliations:** 1 The School of Life Science, Sun Yat-sen University, Guangzhou, China; 2 Faculty of Medicine, Department of Physiology, Chinese University of Hong Kong, Shatin, Hong Kong; University of Cincinnati, United States of America

## Abstract

**Background & Aims:**

Symptoms of constipation are extremely common, especially in the elderly. The present study aim to identify an efficacious treatment strategy for constipation by evaluating the secretion-promoting and laxative effect of a herbal compound, naringenin, on intestinal epithelial anion secretion and a rat constipation model, respectively.

**Methods/Principal Findings:**

In isolated rat colonic crypts, mucosal addition of naringenin (100 µM) elicited a concentration-dependent and sustained increase in the short-circuit current (*I_SC_*), which could be inhibited in Cl^−^ free solution or by bumetanide and DPC (diphenylamine-2-carboxylic acid), but not by DIDS (4, 4′- diisothiocyanatostilbene-2, 2′-disulfonic acid). Naringenin could increase intracellular cAMP content and PKA activity, consisted with that MDL-12330A (N-(Cis-2-phenyl-cyclopentyl) azacyclotridecan-2-imine-hydrochloride) pretreatment reduced the naringenin-induced *I_SC_*. In addition, significant inhibition of the naringenin-induced *I_SC_* by quinidine indicated that basolateral K^+^ channels were involved in maintaining this cAMP-dependent Cl^−^ secretion. Naringenin-evoked whole cell current which exhibited a linear I–V relationship and time-and voltage- independent characteristics was inhibited by DPC, indicating that the cAMP activated Cl^−^ conductance most likely CFTR (cystic fibrosis transmembrane conductance regulator) was involved. In rat constipation model, administration of naringenin restored the level of fecal output, water content and mucus secretion compared to loperamide-administrated group.

**Conclusions:**

Taken together, our data suggest that naringenin could stimulate Cl^−^ secretion in colonic epithelium via a signaling pathway involving cAMP and PKA, hence provide an osmotic force for subsequent colonic fluid secretion by which the laxative effect observed in the rat constipation model. Naringenin appears to be a novel alternative treatment strategy for constipation.

## Introduction

The prevalence of constipation has been reported to be as high as 20%, especially in the elderly. Studies have shown that if people only have a bowel movement every 3–4 days, they are more at risk for having colon cancer, hemorrhoids, irritable bowel disease (IBS), and many other illnesses. Most remedies for constipation focus on modulating the motility of the gastrointestinal (GI) tract, which has been reported with severe side effects. Take examples, cisapride, a first generation of promotility agent [1] was withdrawn because of safety concerns (cardiac arrhythmias) [2]. Tegaserod, a selective 5HT(4) receptor agonist, the most widely used medicine in the treatment of chronic constipation has been reported to cause coronary artery contraction, coronary spasm, and even myocardial infarction [3]. Advances in research on the gastrointestinal physiology and pathology have paved the way for innovative new approaches to the treatment of patients with chronic constipation. Although lubiprostone, which enhances gastrointestinal chloride secretion [4], has extended the therapeutic options available to patients with chronic constipation [5], it still remains many adverse events just like nausea, diarrhea, headache and so on [6]. Little medication for constipation is available for promoting the secretory activity of the GI tract safely.

It is well known that the absorption and secretion of electrolytes and fluid in the intestine are achieved via different ion channels, transporters and pumps that are strategically located on apical and/or basolateral membranes [7]. In particular, epithelial Cl^−^ channels, including the cystic fibrosis transmembrane conductance regulator (CFTR), play vital roles in regulating the secretory activities of electrolytes and fluid across the GI tract, abnormalities of which may result in diarrhea [8]–[10] or constipation [11], [12]. Therefore, drugs with stimulating effects on Cl^−^ secretion may to some extent meliorate the symptom of the latter.

Flavonoids, the most widely distributed phenolic compounds ingested by humans and animals with their regular foods, have been reported to play an important dietary role in protection against chronic diseases such as cancer and cardiovascular diseases; they also have antioxidant and antiproliferative properties [13]–[15]. Interestingly, the effects of certain flavonoids on anion secretion across the intestinal tract *in vitro* have been noted previously [16]–[18]; however, little is known about their use in the treatment of constipation as well as their direct influence on the GI mucosa in *vivo*, where these compounds come in contact with immediately after oral intake,

Naringenin was supposed to be a potent stimulator of chloride secretion in colonic epithelia with significant improvement of symptoms of constipation. The present study examined the effect of flavanone naringenin (NAR, aglycone), a main active flavonol compound extracted from Citrus fruits (oranges and grapefruits) [19], on anion secretion of colonic epithelium and on a rat model of spastic constipation presenting with reduced colonic mucus [20], first indicating its therapeutic potential for treatment of constipation and other relative diseases.

## Results

### NAR-induced Isc response

**Figure 1:**
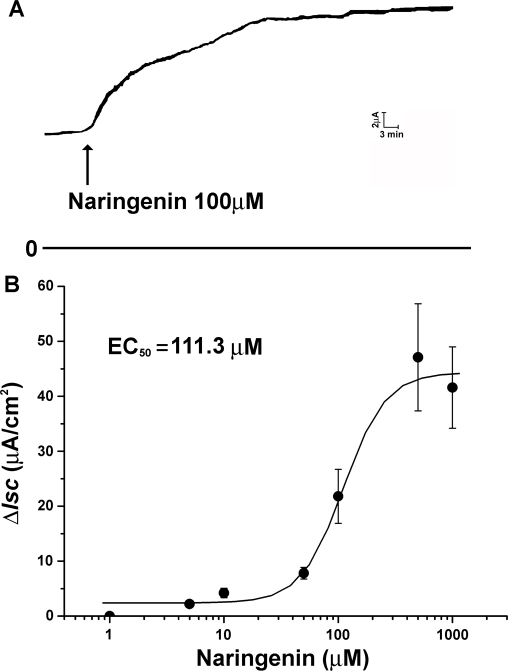
Effect of NAR on anion transport across rat colonic mucosa. (A) NAR 100 µM on basolateral (bl) side resulted in an increase in short circuit current (*I_SC_*), (B) NAR (1 µM–1 mM) stimulated a concentration-dependent short circuit current response (n = 5). Different concentrations of NAR were added to basolateral side. Each data point represents a mean±SE.

The basal *I_SC_* in isolated colonic mucosa were 21±1.2 µA/cm^2^ (n = 20) and basolateral application of NAR (5 µM–1 mM) caused a sustained (Fig. 1A) and concentration-dependent (Fig. 1B) increase in *I_SC_* with an apparent EC_50_ of about 111.3 µM. NAR-induced changes in Isc were calculated as current change for 20 min (µA/cm^2^) (Fig. 1).

### Ionic basis of the NAR-induced I_SC_

**Figure 2 pone-0003348-g002:**
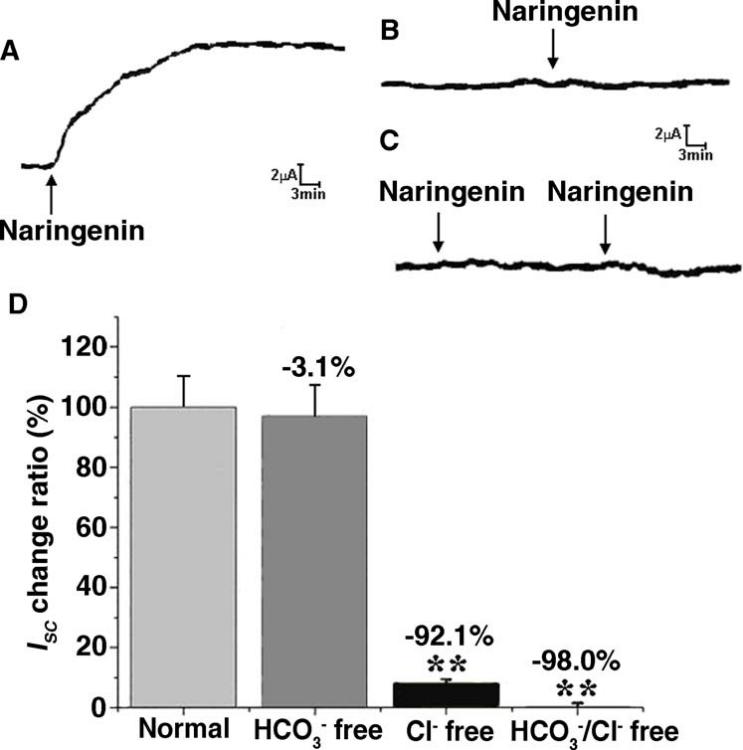
Ionic basis of NAR-evoked *Isc*. Representative *I_SC_* recording with arrows indicating NAR (100 µM) added basolaterally,. Values are mean±SE; ** *P*<0.001. (A) HCO_3_^−^-free K-HS; (B) Cl-free K-HS; (C) Cl^−^/HCO_3_^−^-free K-HS; (D) Comparison of NAR-induced total charges transferred in normal, HCO_3_^−^-free K-HS, Cl^−^ free K-HS and Cl^−^/HCO_3_^−^-free K-HS.

**Figure 3 pone-0003348-g003:**
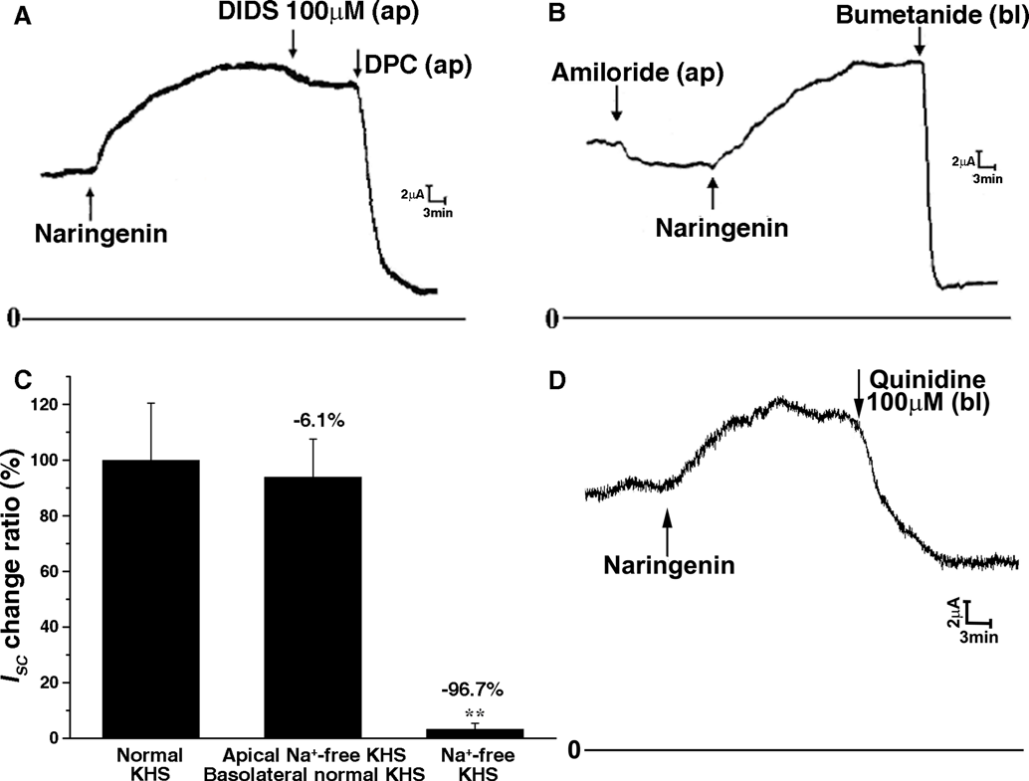
Effect of different channel blockers and bumetanide on *Isc* evoked by 100 µM NAR in rat colonic mucosa. (A) Stimulation with NAR on basolateral side resulted in an increase in *I_SC_*, which is not abolished by DIDS (100 µM, apical), but by apical (ap) application of DPC (1 mM). (B) The NAR induced Isc increases doesn't change when the tissue was pretreated with amiloride (ENaC blocker, 100 µM), but was abolished by basolateral application of 100 µM bumetanide. (C) Comparison of NAR (100 µM)-induced *I_SC_* obtained in control (normal K-H solution), apical Na^+^-free K-H solution with basal normal K-H solution, Na^+^-free K-H solution on both sides. (**P<0.001 *vs.* control). (D) Qunindine (100 µM, bl) inhibited NAR (100 µM, bl) stimulated *I_SC_*.

In order to study the ion species involved in mediating the NAR-induced *I_SC_*, a series of ion substitution experiments were conducted (Fig. 2), and different Cl^−^ channel blockers were examined (Fig. 3A). The change in *I_SC_* was defined as the maximal rise in *I_SC_* following NAR stimulation and it was normalized to current change per unit area of the epithelial monolayer (µA/cm^2^).

DIDS (100 µM, *n* = 4, Fig. 3) added to the mucosal side did not affect the *I_SC_* induced by NAR (100 µM), whereas subsequent application of the Cl^−^ channel blocker DPC completely inhibited the increase in *I_SC_* (n = 4). Removal of HCO_3_^−^ from the bathing solution only slightly suppressed the NAR-evoked *I_SC_* by 3.1% (Fig. 2A), whereas Cl^−^ elimination strongly inhibited the *I_SC_* increase by 92.1%, (*n* = 4, *P*<0.001, Fig.2B); while both extracellular Cl^−^ and HCO_3_^−^ were removed, the NAR-induced *I_SC_* was reduced by 98.0%, (*n* = 4, *P*<0.001) (Fig. 2C). These results suggest that the NAR-induced *I_SC_* response should be mainly contributed by Ca^2+^-independent Cl^−^ secretion.

To test whether electrogenic sodium absorption was involved in the NAR-induced *I_SC_* responses, a Na^+^ channel (ENaC) inhibitor, amiloride (100 µM), was used (Fig. 3B). The responses to NAR were not affected by mucosal addition of amiloride. Further investigation showed that the NAR-induced *I_SC_* was completely abolished by serosal application of bumetanide (a Na^+^ -K^+^ -2Cl^−^ cotransporter inhibitor, 100 µM) (Fig. 3B). Removal of sodium from the mucosal K-H solution did not affect the NAR-induced *I_SC_*, however, when sodium free solution was applied to both sides of the mucosa, the NAR-induced *I_SC_* increase was inhibited by 96.7% (Figure 3C), excluding the participation of mucosal sodium absorption.

Owing to the important role for maintaining the driving force for chloride secretion [21], involvement of basolateral membrane K^+^ channel was also investigated, serosal application of quinidine (100 µM) significantly inhibited the NAR-evoked Isc (Figure 3D), indicating that blocking of basolateral K^+^ channels could also affect Cl^−^ secretion.

### Involvement of cAMP and PKA activity in mediating the effect of NAR

**Figure 4 pone-0003348-g004:**
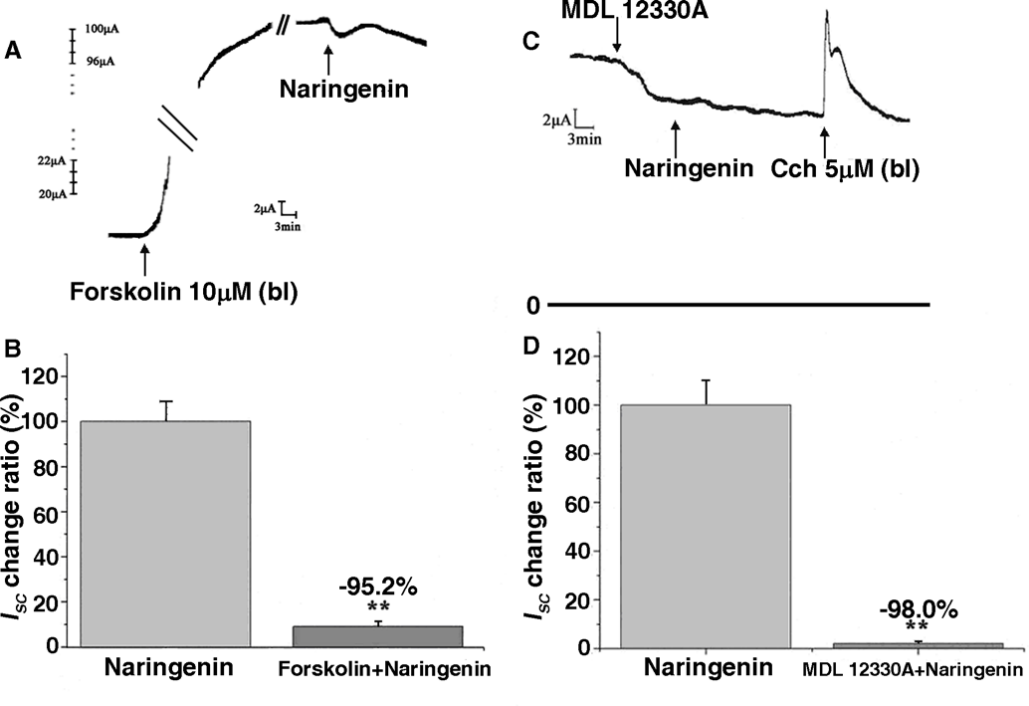
Pretreatment with forskolin reduced NAR–induced *I_SC_* on rat colonic mucosa by 95.2% (A and B) [**P<0.001 *vs.* control]. Pretreatment with MDL-12330A reduced NAR–induced Isc by 98.0% (C and D) [**P<0.001 *vs.* control]. Each column represents the mean±SE (n = 5).

As shown in Fig. 1, NAR caused a long-lasting stimulation on *I_SC_* which is more likely to be a cAMP-mediated short circuit current reaction. In the presence of forskolin (an adenylate cyclase activator), which was used to exhaust the adenylate cyclase so that there will be no further elevation in intracellular cAMP upon subsequent addition of cAMP elevating agents, the NAR induced *I_SC_* increase was almost completely abolished (n = 4, P<0.001, Fig. 4A, B). Coordinately, pretreatment with MDL-12330A (an inhibitor of adenyl cyclase) for 30 minutes dramatically reduced the NAR induced *I_SC_* compared with control (n = 4, P<0.001, Fig. 4C, D).

**Figure 5:**
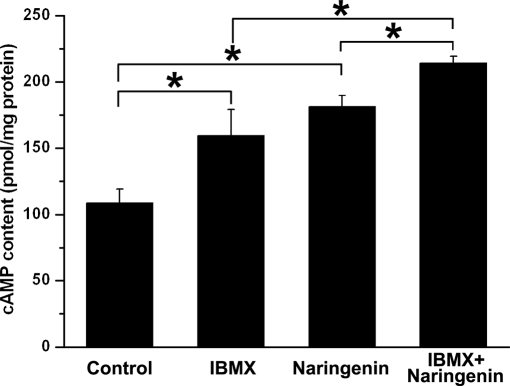
Effect of NAR on intracellular cAMP level of rat colonic mucosa. Comparison of the cAMP generation induced by IBMX (100 µM, both sides), NAR (NAR; 100 µM, mucosal), forskolin (Fors; 0.3 µM, mucosal), each column represents the mean±SE [*P<0.05 *vs.* control].

ELISAs (enzyme linked immunosorbent assay) data presented in Figure 5 confirm that the cAMP dependent pathway was involved in mediating the NAR-induced response. The intracellular cAMP content under basal condition (0.1% dimethyl sulfoxide (DMSO), both sides) was 108.80±10.60 pmol/mg protein (*n* = 3), while after incubation with serosal NAR (100 µM), the cAMP level was 181.34±8.79 pmol/mg protein (*n* = 3). Effect of phosphodiesterase inhibitor IBMX (3-isobutyl-1-methylxanthine, 100 µM) was detected to test whether cAMP degradation contributed to increased cAMP levels, IBMX alone caused an increase in cAMP levels to 159.60±19.68 pmol/mg protein (*n* = 3), whereas incubation with 100 µM NAR in the presence of IBMX (100 µM) led a significant increase in cAMP levels to 214.18±5.00 pmol/mg protein.

**Figure 6:**
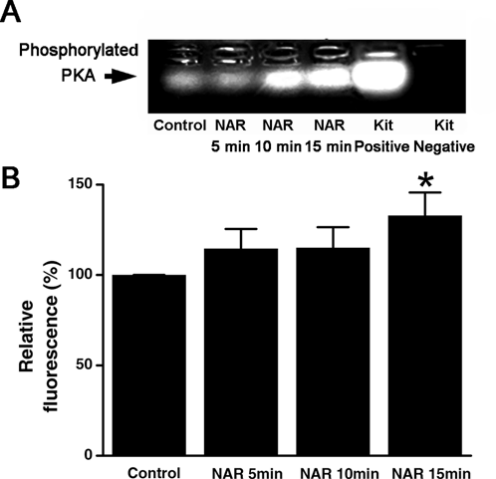
Effect of naringenin on the PKA activity. A, colonic mucosa was treated with DMSO alone (control) or 100 µM naringenin for 5, 10 and 15 min. The positive and negative controls provided by the assay kit are shown in lanes 5 and 6. B, summarized data showing the relative grey value as compared with control. Each column represents the mean±S.E. (*p<0.05).

PKA activity was also measured, as shown in Figure 6, NAR incubation for 5, 10 and especially 15min could increase the PKA activity by 14.63%,15.12% and 32.89%, respectively, compared with which was pretreated with DMSO alone. These results unequivocally demonstrate that NAR could stimulate an increase in cytosolic cAMP content maybe through activating the adenyl cyclase rather than inhibiting the activity of phosphodiesterase, which in turn activate PKA, hence promote Cl^−^ secretion across rat colonic mucosa.

### Characteristic of the NAR-induced whole cell current

**Figure 7:**
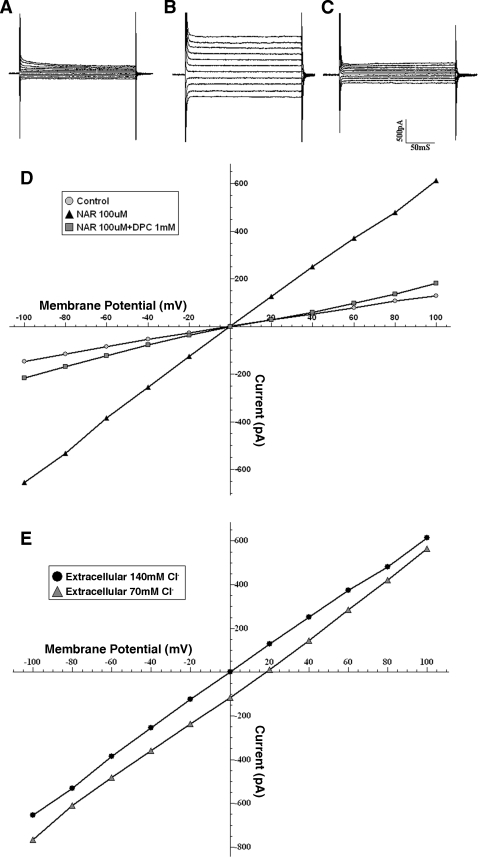
Characteristics of NAR-induced whole cell current in T84 cells. A, B, and C, typical whole cell currents recorded by holding the membrane potential at −30 mV and pulsing to voltages in a range of ±80 mV in 20-mV steps from (A) a control cell; (B) a cell applied with NAR (100 µM) to the bath solution; (C) a cell pre-exposed to 100 µM NAR applied with 1 mM DPC; (D) corresponding current-voltage relationship obtained from currents in A, B and C; (E) Whole-cell recordings obtained after NAR stimulation from a cell bathed in 70 mM NMDGCl with corresponding I–V relationships. Note that reversal potential was shifted from 0 to 17.3 mV, close to the new E_Cl_ = 18.7 mV.

In the whole cell patch clamp experiments, addition of 100 µM NAR resulted in an inward current at holding potential −30 mV. The NAR-activated whole-cell current profile elicited by a series of voltages exhibited time- and voltage-independent characteristics with a linear I–V relationship (Figure 7A–C).The I–V relationships were shown in Figure 6D. The reversal potential of the NAR-induced currents in symmetrical Cl^_^ solutions was close to the Cl^−^ equilibrium, 0 mV. In order to further identify whether the NAR-activated whole-cell currents were mediated by Cl^−^, and not through any nonselective conductance, Cl^−^ concentration in the bath was changed from 140 mM to 70 mM while a pipette containing 140 mM NMDG (N-Methyl-D-Glucamine) -Cl was used. As shown in Figure 6, the reversal potential was shifted to a value 17.3 mV, close to the new theoretic equilibrium value for Cl^−^ 18.7 mV (Figure 7E). The results suggested that currents activated by extracellular naringenin were mediated by Cl^−^. Chloride channel blocker DPC blocked the NAR-activated Cl^−^ current when the membrane potential was held at negative potentials (Figure 7C). This voltage-dependent blockade by DPC was in line with the characteristic of CFTR previously reported in other epithelial cells [22], [23].

### Effect of NAR on rat constipation model

**Figure 8:**
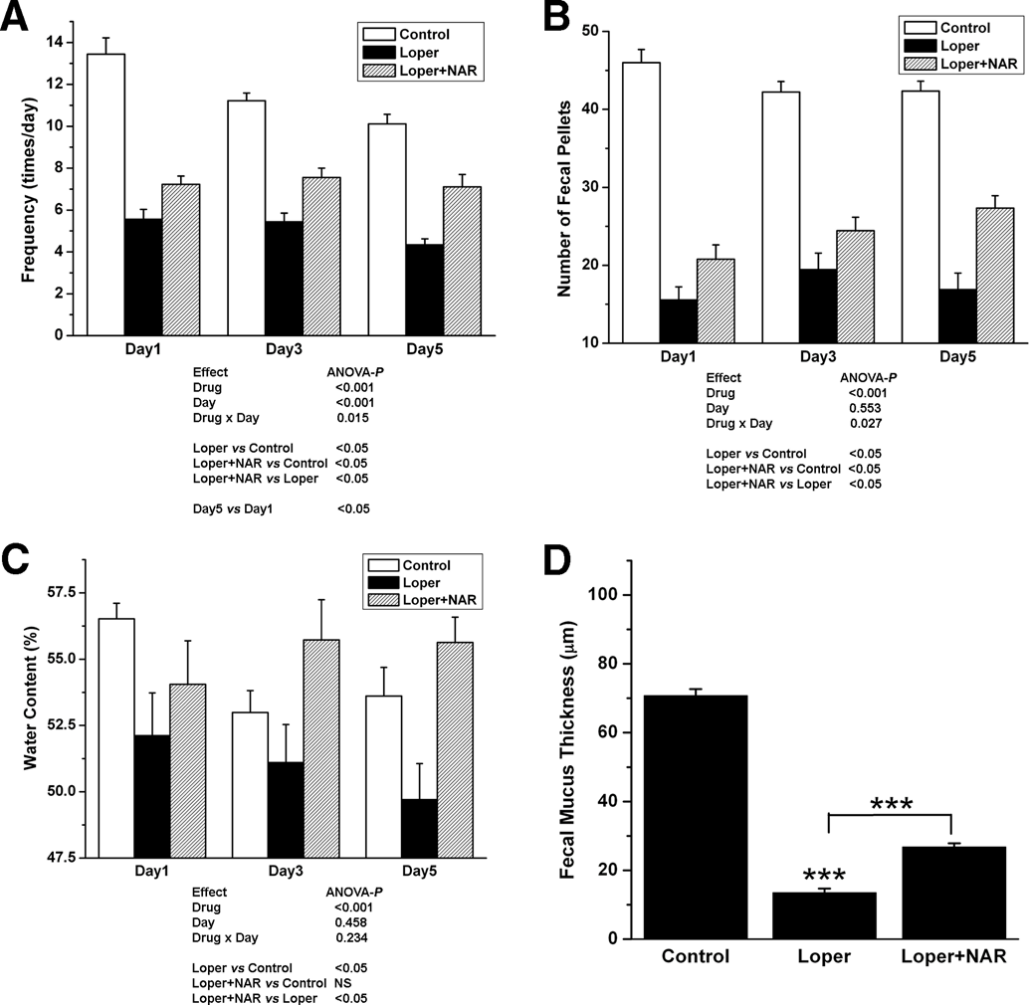
Effect of NAR on the loperamide-induced rat constipation model. (A) Frequency of feces excretion, (B) number, (C) water content (D) Thickness of fecal mucus of control (left bar in each column), loperamide-administered (middle bar in each column, 1.5 mg/kg, twice a day loperamide), and NAR-treated (150 mg/kg, twice a day) loperamide-administered rats (right bar in each column,) on experiment day. Each column represents the mean SE [***P<0.001].

The body weight did not differ significantly between the experimental groups during the experiment. Compared with control group, loperamide markedly reduced the frequency of fecal output (Figure 8A), number of fecal pellet (Figure 8B), fecal water content (Figure 8C) and the average thickness of the mucus layer at the fecal surface (Figure 8D), while all of which were partially restored by NAR with no diarrhea observed.

## Discussion

The present study aimed to identify a rational, efficacious, and ideally cost-effective treatment strategy for constipation. Flavonoids are a group of small molecules derived from plant-based compounds of the common flavone (2-phenyl-g-benzopyrone) structure. Previous study has found that one of the flavonoids, apigenin, could activate CFTR owing to its specific structural components [24]. As CFTR is thought to be the primary pathway for Cl^−^ and fluid transport into the intestinal lumen in cAMP-mediated secretion, there has been a surge of interest in discovering small molecular activators of CFTR that could potentially be used for clinical therapies [25].

The present study has demonstrated for the first time that naringenin (4′, 5, 7-trihydroxyflavone) is able to stimulate cAMP-dependent Cl^−^ secretion across human colonic epithelia and produce laxative effect on loperamide-induced constipation in rats. These effects are primarily unveiled by the results that NAR could activate an immediate and sustained increase in *I_SC_*, which is due largely to an increased Cl^−^ secretion rather than electrogenic Na^+^ absorption. The supporting evidence includes: (i) removal of Cl^−^ from the bathing solution greatly attenuated the NAR-induced *I_SC_*, however, removal of both Cl^−^ and HCO_3_^−^ did not further reduce the *I_SC_* increase to NAR; (ii) Neither Na^+^ ion replacement in the bathing solutions, nor the pretreatment of the colonic mucosa by amiloride (ENaC blocker) had significant effect on the short circuit current induced by NAR. Furthermore, the observation that NAR-induced current was sensitive to both Na^+^-K^+^-2Cl^−^ cotransporter inhibitor bumetanide and Cl^−^ channels blocker DPC suggests that the NAR-induced *I_SC_* is largely due to mucosal Cl^−^ secretion. Since Cl^−^ secretion, coupled with paracellular Na^+^ secretion, is primarily the driving force for fluid secretion across the GI tract, the ability of NAR to promote colonic Cl^−^ secretion indicates its potential use for reduce symptoms of constipation.

It has been clearly established that electrolytes transport stimulated by elevation of intracellular cAMP were largely dependent on a Cl^−^ channel CFTR across wide range of tight epithelia, such as in the airways and the GI tract. In the present study, it is most likely that the long-lasting *I_SC_* response to NAR was mediated through cAMP signaling pathways which then activates the apical CFTR channels and basolateral K^+^ channels. This was substantiated by the inhibitory effect of a K^+^ channel blocker, quinindine (Figure 3D) and MDL12330A (Figure 4C), an inhibitor of adenylate cyclase, on the NAR-induced *I_SC_*. Moreover, after a maximal stimulation of cAMP dependent secretion by forskolin, NAR was not able to further increase the *I_SC_* (Figure 4A), suggesting that both naringenin and forskolin may share a common intracellular second messenger pathway. In addition, ELISA measurement indeed showed that a significant increase in colonic cAMP production could be induced by 100 µM NAR. Pretreatment of colonic mucosa with NAR in the presence of IBMX could further increase the cAMP content compared with IBMX alone, demonstrating the possibility that naringenin might have activated the basolateral adenylyl cyclase rather than inhibited phosphodiesterase. However, further experiments are required to investigate which adenylyl cyclase isoforms account for the effect of naringenin on the generation of these regulatory signals. PKA, which is downstream of cAMP in the signaling pathway, was also activated by naringenin. As elucidated in several studies, CFTR activation is normally regulated by ATP release, elevation of cytosolic cAMP [26] and protein kinase A (PKA). It is therefore that the *I_SC_* response to NAR was most likely mediated by intracellular cAMP production generated by adenylate cyclase, which in turn activates PKA and subsequently the apical cAMP-dependent chloride channel that was probably CFTR. The inhibition of the NAR-induced *I_SC_* by DPC, further demonstrates the involvement of CFTR.

To further confirm whether CFTR was involved in the NAR induced chloride secretion, we also performed whole-cell patch-clamp experiment on T84 cells, with several lines of evidence suggesting that the NAR-activated Cl^−^ current in the T84 cells is characteristic of CFTR [27]–[30]. First, the NAR-activated Cl^−^ current was time and voltage independent and exhibited a linear I–V relationship; Second, as with CFTR, the NAR-activated Cl^−^ current was inhibited by DPC in a voltage-dependent manner (Figure 7B and C) [22], [23]; Additionally, the demonstrated ability of NAR to activate CFTR *in vitro* rules out the therapeutic possibility of naringenin for constipation through stimulating Cl^−^ secretion *in vivo*.

Indeed, in the present study, the results obtained *in vitro* were validated in an *in vivo* loperamide-induced constipation rat model. The salient features of this model include less daily fecal excretion, lower water content and thinner average thickness of mucus of fecal pellets (Figure 8) compared with the control rats. Although previous studies reported that the anti-diarrheal activity of loperamide is mainly due to its inhibitory effect on colonic peristalsis [31] thus loperamide-induced constipation is considered to be a model of spastic constipation [32], our data also suggest that loperamide may inhibit intestinal water secretion as seen in human jejunum [33]. The frequency and number of fecal excretion, water content and the layer of acid mucin secreted by epithelium of the NAR-treated loperamide-administered group was significantly higher than that in the loperamide-administered group, suggesting that NAR produces a laxative effect and alleviate the symptoms of loperamide-induced constipation. Together with the data obtained from rat mucosa and T84 cells, the observed laxative effect on the constipation model is likely to be due to the modulation of electrocytes secretion, rather than intestinal motility, by NAR. Additionally, western blot experiments showed that there was no difference in CFTR expression between the control (data not shown), loperamide and NAR-treated loperamide-administered group, implying the direct modulation of naringenin on the CFTR function rather than expression. The recovery of water content, number of fecal pellets as well as frequency of feces excretion provide convincing evidence for the therapeutic potential value of naringenin in relieving constipation and indicate that the frequency and symptoms of constipation may be reduced by dietary intake of fruits, such as oranges and grapefruits, where rich content of NAR is found.

In closing, we have demonstrated for the first time that a fruit-contained flavonoid, NAR, has potent effect on Cl^−^ secretion in rat distal colon and activation of Cl^−^ conductance in human colonic T84 cells, which is mediated by intracellular cAMP-dependent and PKA involved regulatory pathway. The observed laxative effect in a rat constipation model indicates the possibility that oral administration of naringenin may provide an effective and alternative treatment strategy for prevention and alleviateing symptoms of constipation.

## Materials and Methods

### Tissue Preparation

Rat colonic mucosa were used for short circuit current, cAMP and PKA measurements. Male Sprague-Dawley rats of 200–300 g body weight were killed by CO_2_ asphyxiation according to the guiding principles for the care and use of animals approved by the ethics committee in Sun Yet-sen University. Four pieces of distal colon dissected from each animal were rinsed with ice-cold Phosphate Buffer Solution (PBS), and the muscle layers were separated from the mucosal side by blunt dissection.

### I_SC_ measurements

For all *I_SC_* measurement, tissue samples were cut into appropriate size and then mounted between two halves of modified ussing chambers with an internal area of 0.45 cm^2^. Electrodes for measuring transepthelial potential difference (PD) and passing current were connected to the chamber. The transepithlial PD were clamped at 0 mV, then the short circuit currents were recorded with VCC MC6 voltage-current clamp amplifier (Physiologic instrument, San Diego, CA) and simultaneously displayed via a signal collection and analysis system (BL-420E, Chengdu Technology & Market Co. Ltd, China). The change in *I_SC_* was defined as the maximal rise in *I_SC_* following agonist stimulation and was normalized to current change per unit area of the epithelial monolayer (µA/cm^2^). Transepithelial resistance (Rt) was calculated by measuring the current response to a 1mV pulse. The *I_SC_* value was expressed as positive when the current flow from mucosal to serosal.

### Measurement of intracellular cAMP and Protein Kinase A (PKA) activity

Cytosolic cAMP concentrations were measured by ELISA. After a 30-min period of equilibration in normal KH solution at 37°C, the isolated mucosal sheet was treated with DMSO, IBMX, NAR, and NAR with IBMX, respectively. The cAMP content was assayed using a commercially available enzyme immunoassay kit (Assay Designs, Inc., Ann Arbor, MI). The tissue residue was dissolved in PBS, and protein content was determined using a protein assay kit (Sigma, St. Louis, MO) with bovine serum albumin as the standard. The concentration of cAMP was presented as picomoles per milligram of protein. For PKA activity measurement, the isolated colonic mucosa were incubated with DMSO alone or naringenin for 5, 10 and 15 min and assayed by the PepTag® non-radioactive cAMP-dependent protein kinase assay system (Promega). The phosphorylated and non-phosphorylated samples were separated on a 0.8% agarose gel at 80 V for 30 min. The gel was photographed under UV light and the densitometric analyses of bands were performed using NIH image (obtained from the NIH web sites: Http://rsb.info.nih.gov/nih-image).

### Human colonic T84 cell lines Culture

For whole cell patch clamp study, human colonic T84 cell line obtained from ATCC (American Type Culture Collection) were maintained in Dulbecco's modified Eagle's medium/F-12 supplemented with 10% (v/v) fetal bovine serum, 100 U/ml penicillin, and 100 µg/ml streptomycin. Cells were seeded onto glass coverslips placed in 12-wells culture plates and incubated in a 5% CO_2_ incubator at 37°C for 2 days before patch clamp experiments.

### Whole-cell Patch clamp recording

To isolate single T84 cells, the cells were first placed in Ca^2+^-free D-PBS solution (which consist of: 137 mM NaCl; 8 mM Na_2_HPO_4_; 1.5 mM KH_2_PO4; 2.7 mM KCl) containing 1 mM EGTA (Ethyleneglycotetraacetic acid) for 10–15 min, and then moved to a 1 ml chamber mounted on the stage with immersing lens microscopy system. Signals filtered at 10 KHz were recorded at room temperature by a Multiclamp700A amplifier, Digidata 1322 series interface (AXON Instrument, Foster City, CA) and Pclamp9.0 system (AXON Instrument, Foster City, CA). Patch pipettes were pulled on a horizontal puller P-97 (Sutter instrument Co., Novato, CA) from glass capillary tube with filament and had resistances of 3–7 MΩ. In the episodic recording mode, the voltage was clamped from −100 mV to +80 mV, step +20 mV; while in the gap-free recording mode, the cells were held at −70 mV throughout the period of recording.

### Animal experiments

8-week-old male Sprague–Dawley rats of approximately 250 g body weight were purchased from Guangdong medical laboratorial animals center (Guangzhou China). Rats had free access to a standard diet and drinking water were housed individually in stainless–steel wire–mesh cages in a room maintained at 24±0.5°C, with relative humidity 14±5%, and with a daily photo–period of 08:00–20:00h light. Twenty-seven rats were randomly divided into 3 groups, 9 rats each into loperamide and sodium chloride–administered group, NAR and loperamide–administered and control groups. The administration of loperamide and NAR were carried out after a 2–day adaptation period. Loperamide (1.5 mg/kg, Sigma) and NAR (150 mg/kg, sigma) suspended in 0.9% sodium chloride were orally administrated to rats twice a day at 08:00 and at 20:00 h on experimental days 1–5, while the control rats were only taken 0.9% sodium chloride in the same manner as the experimental rats. Body weight, food intake, fecal water content, fecal excretion frequency and mucus were measured. The excreted feces of individual rats were collected into a dish and their wet weights were measured as immediately as possible after the feces excretion every day during the experimental period. The feces were thoroughly lyophilized and their dry weights were measured. The water content of the fecal pellet was calculated as the difference between the wet and dry weights of the pellet.

Tissue specimens of the distal colon were taken at 10:00 h on the experimental day 5. Segments of the distal colon that included one fecal pellet isolated by ligatures were removed and immediately fixed with 10% formaldehyde. Fixed tissue segments were embedded in paraffin and serially cut into cross sections of 10 µm thick. The sections were stained with alcian blue at pH 2.5 and counterstained with Koelnechtrot. We observed these preparations with a light microscope.

### Solutions and Chemicals

The normal KH solution contained (in mM) 117 NaCl, 4.7 KCl, 1.2 MgSO_4_, 1.2 KH_2_PO_4_, 25 NaHCO_3_, 2.56 CaCl_2_, and 11.1 Glucose. The solution was kept at 37°C gassed with 95% O_2_ and CO_2_ to maintain the pH at 7.4. In Na^+^ free solution, NaCl was replaced with 137 mM NMDG-Cl, NaHCO_3_ was replaced with 10 mM HEPES (4-(2-hydroxyethyl)-1-piperazineethane sulfonic acid). In Cl^−^ free Kreb's solution, NaCl, KCl and CaCl_2_ were replaced with the representive salts of gluconate, other compositions and condition were not altered. In HCO_3_^−^ free solution, HCO_3_^−^ was substituted by 10 mM HEPES, and the solution was bubbled with 100% O_2_. In anion-free solution, the component is the same as Cl^−^-free solution, except NaHCO_3_ was replaced with 20 mM Na-gluconate and 10 mM HEPES, and in the solution the gas was 100% O_2_. In the patch clamp experiments, the pipettes were filled with a solution containing (in mM) 135 NMDGCl, 2 MgCl_2_, 3 MgATP, and10 HEPES, pH adjusted to 7.2 with Tris. The bath solution contained (in mM) 135 NMDGCl, 4.7 CsCl, 2.5 CaCl_2_, 1.2 MgCl_2_, 1.2 NaH_2_PO_4_, 10 Glucose, and 10 HEPES (pH 7.4).

DIDS was obtained from Calbiochem. acetazolamide, amiloride, bumetanide, DPC, naringenin, NMDG, MDL-12330A, and quinidine were obtained from Sigma. Stock solutions of all the chemicals were dissolved in DMSO. Final DMSO concentration never exceeded 0.1% (v/v).

### Data analysis

For Isc measurement, positive currents are defined as those that would be carried by anions moving from the serosal to mucosal compartments and are shown as upward deflections of the traces. Changes in ion transport (*I_SC_*) are given as peak values. Student's t-test (Figure 1–5) or Tukey's multiple comparison after a two-way analysis of variance (ANOVA) (experimental group x day) (Figure 7) were adopted. All the results are expressed as mean±SE. Difference were considered significant at error probabilities smaller than 0.05.
